# Powder Processing in Pharmaceutical Applications—In-Depth Understanding and Modelling

**DOI:** 10.3390/pharmaceutics13020128

**Published:** 2021-01-20

**Authors:** Jan Henrik Finke, Arno Kwade

**Affiliations:** 1 Institute for Particle Technology, TU Braunschweig, Volkmaroder Str. 5, 38104 Braunschweig, Germany; jan.finke@tu-braunschweig.de; 2 Center of Pharmaceutical Engineering—PVZ, TU Braunschweig, Franz-Liszt-Str. 35A, 38106 Braunschweig, Germany

In all production processes of solid dosage forms, powders with a multitude of distributed properties must be processed. Starting with their handling for dispensing and weighing, over blending, dosing, comminution, and granulation, up to capsule filling or tableting, powder properties crucially determine the process performance and product quality. In any case, each powder in pharmaceutical formulations brings along distributions of various microscopic properties, such as particle size, morphology, and surface energies, as well as macroscopic properties, such as flow behavior and bulk densities. Along the process chain, multiple powders are blended or more fundamentally change their properties, e.g., by granulation, causing complex changes that will affect their behavior in subsequent processes. Additionally, transportation and packaging of intermediates and final products can cause changes due to aeration, abrasion, agglomeration or compaction. The determination of powder properties such as particle characteristics, surface structure, and flow properties requires sophisticated, methodologic approaches to measure meaningful parameters under representative conditions. The deep knowledge of powder properties and quick and reliable measurement techniques become a prerequisite in the context of continuous process chains that need to immediately respond to material property changes to keep processes and product quality stable within specified ranges. To achieve this, well-developed model approaches need to be derived to predict and control any complex powder processing operation. This Special Issue is dedicated to in-depth studies presenting fundamental advancement in the scientific field of powder processing in pharmaceutical applications. Contributions address thorough insight into unit operations using model approaches that provide predictions of effects such as overmixing, applied flow characteristics, and residence time distribution based on material properties and process parameters.

The contributions in this Special Issue address powder processing in granulation [1,2], capsule filling [3], tableting [4,5,6,7], and along whole process chains [8], which powders mostly experience in industrial applications. Furthermore, articles span the application depths from scaling from lab to production scale [1,4] and transforming suspensions into dry products [2,8], over in-depth description of sub processes on rotary presses, which depend on powder properties [5,6,7], to statistical experimental design [1,3] and application of artificial neural networks [3].

Granulation processes are addressed for the downstream processing of drug nanosuspensions into dry, flowable products that maintain the nanoparticulate state of the drug [2]. In this context, Raman microscopy was applied to characterize the thickness of the drug-containing layer to better explain differing efficiencies of the embedding of nanoparticles based on carrier solubility. Additionally, the granulation of a plant extract is used to demonstrate a quality by design approach to identify crucial granulation factors and facilitate the scaling of high shear wet granulation on a rational basis, applying a polynomial regression model [1].

Capsule filling with uncoated and coated pellets of different sizes and densities is studied to develop and compare models based on design of experiments and artificial neural network approaches [3]. In this case, artificial neural networks outperform multiple linear regression approaches by far in the prediction of many parameters, e.g., the practically highly relevant fill weight variation.

Powder processing to the most common dosage form of tablets is also most addressed in this this Special Issue. The relevance of the effects of powder properties on various sub-processes that take place on rotary presses is presented in detail. Besides presenting methods to produce tracers with the properties of the investigated powder, the influence of different setups and scales of paddle feeders integrated in rotary presses, as well as of powder properties on the residence time distribution within rotary presses, is evaluated [7]. The subsequent sub-process of die filling is characterized in a comparable way [6]. Effects of insufficient die filling and powder densification are described and differentiated regarding the effects of powder properties and process parameters on these. Within the die, the powder is processed to a compact by compression. This process is studied in depth with powders showing different deformation behaviors, identifying the challenge of calculating negative porosities if the change in solid density under high stresses remains neglected. Thus, a powder compression model taking this solid compressibility into account and fitting it is developed and compared against literature models [5].

A more general perspective on the production process of tablets is provided by a comprehensive study of the scalability of tableting processes between a compaction simulator and rotary presses [4]. The visco-plastic properties of starch were used to identify the slight differences between the compression profiles while the susceptibility of microcrystalline cellulose towards overlubrication was deployed to identify differences between the process scales and to propose an enhanced shear number model that is applicable also to compaction simulators.

A whole process chain from transfer of suspension to dry powder by freeze drying and comminution through a sieve over blending with tableting excipients to compaction is studied to evaluate the influence of these several process steps on the viability of microorganisms [8]. This study aiming at probiotic tablet formulations provides an approach to achieve a high survival rate of the microorganisms by the correct choice of excipients and process parameters.

Altogether, the articles of this Special Issue stress the importance of powder processing in pharmaceutical applications and the contribution of in-depth process understanding and modelling of correlations towards the development approaches that provide predictive power to safely and reliably handle such complex systems as powder blends. Finally, these can enable us to develop new drugs—and by that, in most cases, new powders—more quickly and based on scientific rationales into products, allowing for an earlier entrance into the market.
